# The Effects of Combined Treatment with Naringin and Treadmill Exercise on Osteoporosis in Ovariectomized Rats

**DOI:** 10.1038/srep13009

**Published:** 2015-08-11

**Authors:** Xiaolei SUN, LI Fengbo, MA Xinlong, MA Jianxiong, Bin ZHAO, Yang ZHANG, LI Yanjun, LV Jianwei, Xinmin MENG

**Affiliations:** 1 Orthopedics Department, Tianjin Hospital, NO.122 Munan Road Heping District, Tianjin 300050, China

## Abstract

Osteoporosis is a disease characterized by low bone mass and progressive destruction of bone microstructure, resulting in increased the risk of fracture. Previous studies have demonstrated the effect of naringin (NG) or treadmill exercise (EX) on osteoporosis, however, reports about effects of NG plus EX on osteoporosis are limited. This study was designed to investigate the impact of combined treatment with naringin and treadmill exercise on osteoporosis in ovariectomized (OVX) rats. Three months after bilateral ovariectomy, Seventy-five rats were randomly assigned to the following treatment groups: OVX, sham-operated (SHAM), NG, EX, or NG plus EX treatment. Treatments were administered for 60 days. Bone metabolism, bone mineral density, trabecular bone parameters, immunohistochemistry, and the bone strength were evaluated. Compared to the OVX groups, all treatments increased bone volume (BV/TV), trabecula number (Tb.N), trabecula thickness (Tb.Th), bone mineral density (BMD), and mechanical strength. NG + EX showed the strongest effects on BV/TV, Tb.Th, and biomechanical strength. Additionally, decreased C-terminal telopeptides of type I collagen (CTX-1) and enhanced osteocalcin (OCN) expression were observed in the NG + EX group. The present study demonstrates that the NG + EX may have a therapeutic advantage over each monotherapy for the treatment of osteoporosis.

Osteoporosis is a common disease worldwide, and characterized by decreased bone mineral density and progressive destruction of bone microstructure, resulting in an increased bone fragility and risk of fracture^1^. There are many factors that contribute to osteoporosis, such as estrogen deficiency, hereditary, nutritional deficiencies, chronic diseases and aging. An especially common culprit is estrogen deficiency which has been linked to the development of osteoporosis in postmenopausal women. In a healthy subject, bone remodeling is maintained by the strictly coupled activities of bone-forming osteoblasts and bone-resorbing osteoclasts^2^. Estrogen deficiency caused by menopause contributes to increased bone turnover and osteoclastic resorption, exceeding the rate of osteoblastic formation and results in a loss of bone mass and decreased bone strength in women^3^.Many pharmaceutical treatments have been developed to help prevent bone loss and increase bone mass, such as antiresorptive drugs (estrogen, calcitonin, bisphosphonates) and callus formation drugs (parathyroid hormone). However, these therapies have low long-term compliance and side effects and are cost prohibitive^4^,^5^. Therefore, scientists began to study alternatives such as natural remedies, which are thought to be healthier and safer treatments.

Naringin is a polymethoxylated flavonoid, the main active compound of traditional Chinese medicine Rhizoma drynariae, and possesses a variety of biological and pharmacological effects. Naringin acts as anti-inflammatory, anti-mutagenic, analgesic and antioxidant^6^. Studies have reported that naringin also promotes the proliferation of rat osteoblast-like cells (UMR-106), mouse osteoblastic cells and human mesenchymal stem cell differentiatio^6^,^7^,^8^. In addition, animal studies have shown that naringin increased bone mineral density, bone mass, mechanical strength of the callus and other produced other beneficial effects in ovariectomized (OVX)-induced mice^6^,^9^. Studies have also suggested that naringin may decrease the level of plasma cholesterol via hepatic HMG-CoA reductase activity suppression and fecal sterol excretion increase^10^.

Similarly, exercise is considered to have positive influence on bone morphology and the mechanical loading generated by physical activity has been speculated to aid in preservation and improvement of bone mass and strength^11^. Furthermore, studies have reported that osteoblasts and osteoclasts sense and respond to mechanical stimuli and that mechanical stimulation increased osteoblastic release of *ALP*^12^, nitric oxide (*NO*)^13^, prostaglandin E_2_ (*PGE*_*2*_)^14^, and regulates Runx2 activation^15^. Additionally, studies have reported that mechanical stress may inhibit osteoclast differentiation and the activity of bone resorption^16^. Although several studies have demonstrated a stimulatory effect of exercise on bone tissue, it is not recommended as a substitute for medical treatment^17^.

There are few published studies investigating the effects of the combination therapy - physical exercise and drugs - for treatment of osteoporosis. The purpose of this study was to evaluate the effect of naringin combined with treadmill exercise on osteoporosis in ovariectomized (OVX) rats. After sixty days of treatment, the systemic bone metabolism condition and bilateral femur were evaluated by serum analysis, histology, histological and fluorescent analysis, immunohistochemistry, dual energy X-ray absorptiometry (DEXA), biomechanics, and micro-computed tomography (micro-CT) assessment. These measurements were performed to investigate the effects of different treatments on osteoporosis in OVX rats.

## Materials and Methods

All experimental procedures were approved by the Institutional Animal Care and Use Committee at the Tianjin Hospital Ethics Committee of China. The methods were carried out in accordance with the approved guidelines.

### Animals

Healthy 3-month-old Female Sprague-Dawley (body weight, 230 ± 10 g) was provided by the Experimental Animal Center of the Tianjin Hospital. The experimental animals received humane care. The animals were housed in an air-conditioned environment (22 ± 2 °C), with a 12-h light/dark cycle (7:00–19:00) and were allowed free access to food pellets and water throughout the experiment.

Seventy-five rats were randomly divided into five groups (15/group), and four groups were removal of the bilateral ovaries (OVX). The rats in the sham group were incision and sutured without removal of the ovaries. Three months after post-surgery, the four OVX group treated with vehicle, naringin (NG), treadmill exercise (EX) ,and naringin plus treadmill exercise (NG + EX) for 60 days. The sham group was orally treated with H_2_O only. In order to obtain dynamic parameters of callus formation and remodeling, tetracycline (25 mg/kg) were injected intramuscular at 10 and 3 days before sacrifice. On the last day of treatment, blood was collected from the heart under ketamine anesthesia. After centrifugation, serum was obtained and kept at −80 °C until further analysis. Femur were dissected and 5 right femoral in each group fixed with 75% ethanol stored at 4 °C for undecalcification and 5 right femoral in each group fixed with 4% paraformaldehyde, the remaining femur with phosphate buffered saline stored at −20 °C until analysis of BMD, Micro-CT and femoral mechanical test.

### Naringin intervention

Naringin was purchased from Xi’an Guanyu Bio-tech Company (Xi’an, China). The purity was over 98%. For the *in vivo* study, naringin was dissolved in 0.9% saline and concentrations of 300 mg/ml were prepared. NG and NG + EX group rats were gavaged once per day with 300 mg/kg naringin which was delivered in 0.9% saline. The OVX and sham group were orally treated with the same amount H_2_O only.

### Exercise intervention

Rats allocated to EX and NG + EX group were exercised on a motorized treadmill five consecutive days/week at a 5% incline for 60 days. Rats ran in individual running compartments that contained an electronic grid attached at the base of each compartment. Rats were introduced to the treadmill-training protocol over a 1-week period, during which the speed was gradually increased from 10 to 20 m/min, and the duration of each session increased from 10 to 60 min/session. For the remaining 53 days, rats ran at 22–24 m/min for 60 min/session^18^. Animals in all groups were allowed free cage activity when not exercising.

### BMD and Micro-CT analysis

The BMD of right femur in each group (5/group) were measured by dual-energy X-ray absorptiometry (DEXA) (LUNAR DPXIQ, GE Healthcare, USA). After DEXA measurement, the trabecular bone microarchitecture of the distal right femoral metaphysis was measured using a microtomography scanner (Sky Scan 1076, Kontizh, Belgium), with a slice thickness of 21 μm and the voxel resolution of 22 μm^3^ .The volume of interest (VOI) was selected as a region twenty-five slices away from the distal femur growth plate to 125 slices. The 3D images were obtained for visualization and display. Bone morphometric parameters including bone volume over total volume (BV/TV), trabecula number (Tb.N) and trabecula thickness (Tb.Th) were obtained by analyzing the VOI. The operator conducting the scan analysis was blinded to the treatments associated with the specimen.

### Histological and fluorescent analysis

After fixation in 75% alcohol for 7 days, the right femur (5/group) specimens were dehydrated in graded ethanol and embedded in methylmethacrylate (Technovit 7200 VCL; Exact Apparat-bau, Nordenstedt, Germany) without decalcification. Seven μm-thick sections were obtained along the sagittal plane of the distal femur using tungsten carbide blades (Leica SP1600, Germany). Finally, sections were stained in giemsa for observation. The parameters were analyzed by Image-Pro Plus 6.0 software.

### Mechanical testing

The mechanical strength of the shaft and neck of the left femur (10/group) was measured with the method described by Ma and Fu^19^. A vertical load was applied to the left mid shaft of the femur and the top of the left femoral head from Endura TELELF 3200 mechanical testing instruments (Bose Corporation, USA). Mechanical loading speed at a constant displacement speed of 5 mm/min until the mid-shaft of the femur and femoral neck fractured. The fracture load was recorded.

### Osteocalcin and *CTX-1* measurements

Osteocalcin (OCN) is a small noncollagenous matrix protein of the bone that derives from new cellular synthesis and a specific marker of bone formation whenever formation and resorption are uncoupled. Increased serum OCN reflects the acceleration of osteoblastic activity and bone turnover^20^. Bone consists of a calcified organic matrix, which is composed of 90% type I collagen^21^. During bone resorption, type I collagen is degraded, and small fragments are liberated into the blood stream. Higher C-terminal cross-linked telopeptides of collagen type I (CTX-1) levels are associated with lower bone mineral density values in osteoporosis^22^. Serum levels of OC and CTX-1 were assayed using ELISA assay kit (blue gene, China) according to the manufacturer’s instruction, and serum samples from the heart were analyzed (n = 6/group).

### Immunohistochemistry

For immunohistochemical evaluation of OCN and CTX-1 expression (5/group), specimens were fixed in 4% buffered formaldehyde for 2 days at room temperature, and then decalcified in ethylenediaminetetraacetic acid (changed every 3 days) for 4 weeks. Decalcified tissues were then washed, dehydrated in gradient alcohol, embedded in paraffin wax, and cut into 4-μm-thick sections along the sagittal plane of the distal femur. Immunohistochemical localization of OCN and CTX-1 was carried out using commercially available antibodies according to the manufacturer’s suggested protocol (Sigma Company, USA). Negative controls were obtained by omitting the primary antibody. Stained sections were examined qualitatively under light microscopy (Nikon, Japan) with a digital camera.

### Statistical analysis

Data were expressed as mean ± standard deviation (SD). Statistical analyses were performed using the statistics package SPSS16.0 (SPSS, Chicago, IL, USA). Multiple comparisons between groups were carried out using one-way ANOVA and Tukey’s post hoc test. P-values < 0.05 were considered statistically significant.

## Results

All the rats recovered well after the surgery procedure. No animal deaths and weight loss were observed during the treatment period.

### BMD and Micro-CT analysis

**Figure 1:**
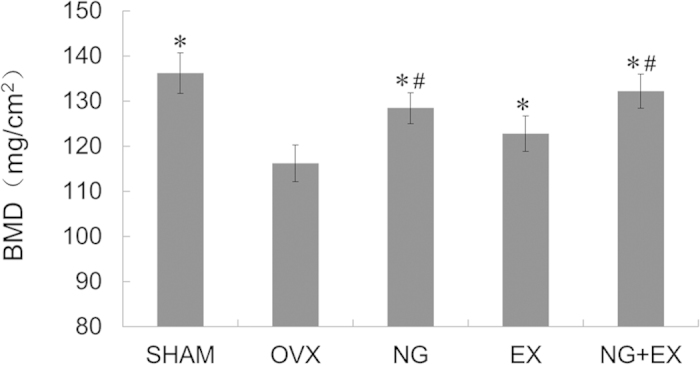
Result of BMD by DEXA examination. Values are means ± Standard deviation, n = 5. *p < 0.05 vs. OVX group; ^#^*p* < 0.05 vs. EX group as evaluated by ANOVA.

Results of right femoral BMD by DEXA were presented in Fig. 1. All the treated groups showed significantly increased BMD than the OVX (*p *< 0.05). NG and NG + EX showed similar BMD of SHAM, but both were higher than that of EX (*p *< 0.05).

**Figure 2:**
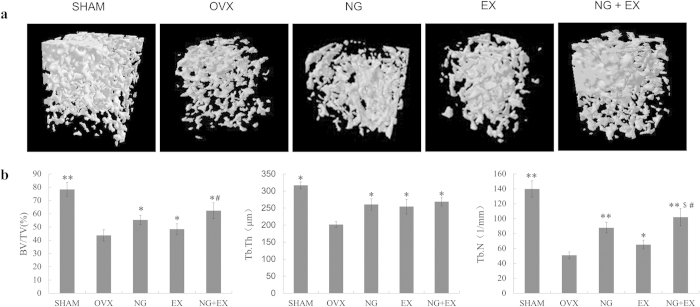
(**a**) Representative sample from each group: 3D architecture of trabecular bone within the distal femoral metaphyseal region. (**b**) Effects of naringin or exercise on the trabecular bone volume, number of trabeculae, and thickness of the trabeculae of the distal femoral metaphysic in OVX rats by microtomography analysis. Values are means ± Standard deviation, n = 5. *p < 0.05, **p < 0.01 vs. OVX group; ^#^*p *< 0.01 vs EX group; ^$^*p *< 0.05 vs. NG group as evaluated by ANOVA.

Three-dimensional images of the right distal femur with differences in trabecular microarchitecture among the five groups are presented in Fig. 2a. Analyses of the data from the right distal femur revealed that OVX rats had significant (*p *< 0.01) lower trabecular BV/TV, Tb.N and Tb.Th, as compared to the sham rats. NG and NG + EX showed increased BV/TV than the VOX and EX (*p *< 0.05), while no difference in Tb.Th was observed between treated groups (*p* > 0.05). Moreover, NG + EX treatment presented the strongest effect on Tb.N than EX (*p *< 0.05, Fig. 2b).

### Histological and fluorescent analysis

**Figure 3:**
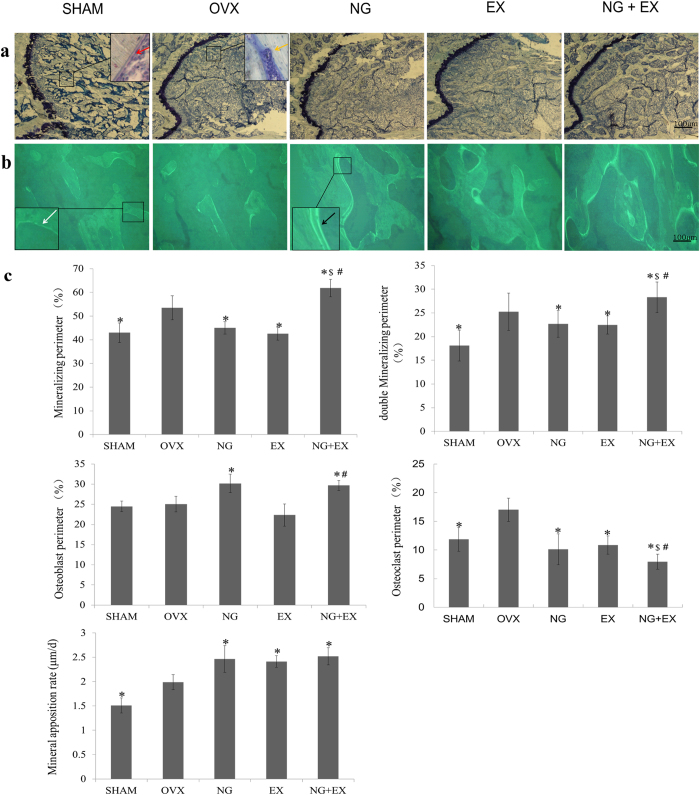
(**a**) Undecalcified histological sections, stained with giemsa, of the sagittal plane through the distal of femur (magnification, ×100); the *scale bar* represents 100 μm. The red arrows show the osteoblast perimeters, visible aligned osteoblasts. The yellow arrows show the osteoclast perimeters, visible absorption of osteoclasts. (**b)** Undecalcified sections observed under a laser confocal scanning microscope showing the tetracycline fluorescence (magnification, ×100); the *scale bar* represents 100 μm. The white arrows show the single mineralizing (single labeled). The black arrows show the double mineralizing (double labeled). (**c**) Quantitative results of the mineralizing perimeter, double mineralizing perimeter, osteoblast perimeter, osteoclast perimeter and MAR, n = 5 specimens/group. Values are mean ± Standard deviation, n = 5. *p < 0.05, OVX group; ^#^*p *< 0.01, vs EX group; ^$^*p *< 0.05, NG group as evaluated by ANOVA.

Undecalcified histological and tetracycline fluorescence images were exhibited in Fig. 3a,b. After treated with naringin and/or treadmill exercise for 60 days, more trabecular were observed in the NG + EX group compared to NG or EX alone. NG and NG + EX showed the similar effect on osteoblast perimeter. All treatments increased mineral opposition rate (MAR), but no significant difference was observed between treated groups.

In fluorescent analysis (Fig. 3c), NG + EX showed the largest mineralizing perimeter (*p *< 0.05). NG, EX, and NG + EX showed similar effect on MAR, which was increased by 25.9%, 23.2%, and 28.7% compared to the VOX, respectively (*p *< 0.05). NG + EX showed the strongest effect on osteoclast perimeter (*p *< 0.05). NG and NG + EX showed similar osteoblast perimeter, while NG alone showed increased osteoblast perimeter and decreased osteoclast perimeter levels compared to the OVX group.

### Mechanical testing

**Figure 4:**
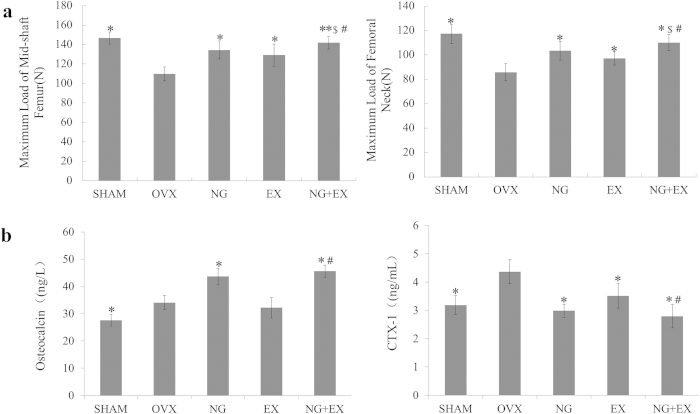
(**a**) Biomechanical results expressed as maximum load of mid-shaft femur and femoral neck, n = 10 specimens/group. (**b**) Results of the serum analysis of OCN and CTX-1, n = 6 samples/group. Values are mean ± Standard deviation. *p < 0.05, **p < 0.05 vs. OVX group; ^#^*p *< 0.05, vs. EX group; ^$^*p *< 0.05, vs. NG grou*p* as evaluated by ANOVA.

The average maximum fracture loading to the left femoral neck and femoral mid-shaft was lower in the OVX control group as compared with the naringin and EX groups. The strongest effects on strength of femoral were observed in the NG + EX group (*p *< 0.05). Compared to the OVX group, NG, EX, and NG + EX groups increases of the average maximum fracture loading of left femoral neck were 20.4%, 13.1%, and 28.3%. NG, EX, and NG + EX group increases of the average maximum fracture loading of right femoral mid-shaft were 22.4%, 17.7%, and 29.3% (*p *< 0.05, Fig. 4a).

### Serum analysis

Serum levels of OCN and CTX-1 were measured to provide an evaluation of bone formation and resorption activity under NG and/or EX treatment (Fig. 4b). NG and NG + EX increased in OCN levels by 28.3% and 33.7%, respectively (*p *< 0.05); while EX displayed similar OCN levels when compared to the VOX control (*p *> 0.05). For analyses of CTX-1 levels of NG, EX, and NG + EX decreased by 31.7%, 19.4%, and 36.1% compared to the VOX control (*p *< 0.05).

### Immunohistochemical evaluation of OCN and CTX-1

**Figure 5:**
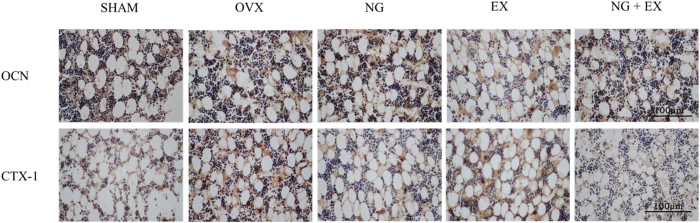
Immunohistochemical localization and reactivity of OCN and CTX-1 in the distal femur (magnification, ×400); the *scale bar* represents 100 μm. Control, naringin and exercise intervention grouptreated groups and test items were presented in the figure.

Immunohistochemical reactivity images for OCN and CTX-1 were exhibited in Fig. 5. Strong reactivity of OCN was observed in the OVX and NG groups, and significant inhibited in the EX group. NG + EX showed no significant difference reactivity of OCN than the OVX. While OVX displayed markedly high CTX-1 levels when compared to the SHAM group. All intervention groups significantly reduce CTX-1 expression in comparison to the OVX group. Among them, NG + EX resulted in the greatest decrease in CTX-1 expression. This result indicated that NG, NG + EX revealed inhibited CTX-1 and enhanced OCN expression simultaneously. While EX showed reduce both OCN and CTX-1.

## Discussion

In the current study, we found that NG + EX exhibited greater therapeutic effects on osteoporosis than either monotherapy in OVX rats, with the highest values of BV/TV and Tb.N in micro-CT evaluation, significantly increased OCN and decreased CTX-1 levels identified in serum analysis, the strongest effects on fluorescence-marked rate and osteoclast perimeter in fluorescent analysis, and the strongest effects on femoral neck and femoral mid-shaft strength in biomechanical tests. These results indicated that the combined treatment of naringin and treadmill exercise had an additive effect on osteoporosis reduction in OVX rats.

In this study, we used ovariectomized rats with the initial age of 3 months as a postmenopausal model because these rats are reproductively mature and capable of responding appropriately to estrogen deficiency and its sequela following ovariectomy. Three months were allowed to pass after surgery for the establishment of standard osteoporotic animal models as validated by literature^23^. Moreover, the characteristics of the bone loss were similar to those of postmenopausal osteoporosis in women. The results indicated that trabecular bone mass and BMD were lower in OVX than in SHAM rats. Bone turnover was increased by estrogen deficiency, as evidenced by higher serum OCN and CTX-1 as well as higher MAR in OVX than in SHAM rats. These findings are in agreement with the results of the previous studies^23^,^24^. All the treated groups showed significantly increased BMD than the OVX control, but no significant difference was found between NG + EX and NG. Although BMD was recognized as an important predictor of osteoporosis related fractures, studies have shown that when BMD criteria is used to diagnose the chance of osteoporotic fractures, only 10%–53% of fractures are actually attributed to BMD^25^. Micro-CT voxel based test unit can be used to detect lesions and structure in bone early^26^ and micro-CT scan results showed that NG + EX show the strongest effect on BV/TV, Tb.Th, Tb.N. These result demonstrated that NG + EX had an additive or synergistic effect on bone mass and microarchitecture of trabecular bone in OVX rats.

A three-point bending test and the femoral neck compression test was performed to evaluate the effect of NG + EX on the mechanical strength of the femoral shaft and femoral neck. The three-point bending test is a conducive test for cortical regions, and the femoral neck is the most important site for fractures in osteoporosis^27^. We found that NG plus EX demonstrated greater strength than those of the monotherapy treatment group. It is also worth noting that the combined group had a femoral neck and mid-shaft strength similar to the SHAM group.

Undecalcified histologic sections are widely used to evaluate dynamic microstructural parameters of bone. Tetracycline is able to specifically bind calcium used in decalcification and thus be used to measure multiple parameters of bone tissue dynamics. According to the American Society for Bone and Mineral Research (ASBMR) proposed standard nomenclature^28^. Double tetracycline fluorescence labeling, under a fluorescence microscope, we can see the tag line for the first time nearly buried in the bone matrix in the trabecular surface, the second marker closer to the bone surface. The tetracyclines labeling rate reflects the rate and amount of new bone formation. The distance between two lines of fluorescently labeled tetracycline was the amount of new bone formation at the time of the two marks. Osteoclastic resorption surfaces include two cases. One is absorbed by osteoclast on trabecular bone surface. Another is bone absorption has been completed, osteoclasts have disappeared and osteoblasts has not yet appeared. Osteoclast perimeters were the rate of osteoclastic resorption surfaces/Trabecular bone surface, represents the activity of bone resorption. Osteoblastic surfaces were bone trabecular tissue surface covered with neatly arranged osteoblasts. Osteoclast perimeters were the percentage of osteoblastic surfaces surfaces/Trabecular bone surface, displayed the activity of bone formation. MAR was commonly used to calculate the mean rate of bone formation. MAR obtain by measuring the average of vertical distance between two times tetracycline fluorescence marked lines and the time of interval mark in animal. MAR (μm/d) = N times the average of vertical distance of dual-labeled line/the time of interval mark.

In the present study, EX showed lower levels of osteoblast and osteoclast activity along the perimeter of bone in OVX rats. These findings are in agreement with the results of the previous studies^29^. NG showed increased osteoblast activity, decreased osteoclast activity and more tetracycline-marked bone trabeculae, NG + EX produced the strongest effect on tetracycline-marked bone trabeculae than either monotherapy. NG + EX had an additive effect on osteoclast activity, but osteoblast activity is similar in NG + EX with the NG treatment group. There seemed to be an additive effect of NG + EX on resorption surface and bone mineralization, but this hypothesis was not supported by MAR as all treatments showed similar effects.

Loss of bone mass was typically accompanied by a significant increase in bone remodeling, as was evidenced by altered levels of the bone turnover markers OCN and CTX-1^25^. In a previous study, plasma activity of CTX-1 and the OCN markers of osteoclasts osteoblasts respectively demonstrated significantly greater levels in the OVX group than in the sham-operated group^25^,^30^. A similar phenomenon was observed in the present study through the analysis of serum bone metabolic markers. NG alone showed increased OCN and decreased CTX-1 levels compared to the OVX treatment group. These results suggest that naringin ameliorated bone loss induced by OVX by inhibiting bone resorption and enhancing bone formation. Previous studies have reported that naringin increases the number of osteoblasts and alkaline phosphatase (*ALP)* activity of bone mesenchymal stem cells (BMSCs*) in vitro*^31^. Naringin also decreases the number of osteoclasts *in vitro*^32^. While OCN and CTX-1 were lower in the exercised treatment group than in OVX rats, implying that bone turnover was lower in EX, NG + EX showed decreased CTX-1 levels and similar OCN levels with NG. These results indicate that NG + EX had an additive effect on inhibited bone resorption but did not promote bone formation.

The mechanism of NG + EX is an interesting subject. The NG + EX treatment demonstrated the strongest effect on bone mass preservation and bone strength in OVX rats. Estrogen deficiency-induced osteoporosis belongs to a high conversion type in which bone formation and bone resorption are all increased, but bone resorption is more remarkable^33^. Wiren and Oreffo *et al.* have demonstrated that estrogen receptors are located on the membrane surface of both osteoblasts and osteoclasts; however, estrogen functionality in bone resorption is inhibited and decreases the number of bone remodeling units^34^,^35^. The potential estrogen-like effect of naringin is reportedly capable of competitive binding to estrogen receptors (ERs), and showed potential as a replacement for estrogen in clinical use in lieu of estrogen replacement therapy (ERT)^36^.

In addition, it has been confirmed that osteoblasts and osteoclasts sense and respond to mechanical stimuli^37^. The findings of our current and previous studies are in agreement with Frost’s hypothesis on mechanical loading and the adaptation responses of bone^29^,^38^. Frost suggested that mechanical loads can be expressed as the strains they generate in bone. Strains control bone modeling and remodeling, and thus influence bone mass. Studies have confirmed that appropriate mechanical stimulation promotes the proliferation and differentiation of osteoblasts and inhibit differentiation and bone resorption activity of osteoclasts *in vitro*^16^,^39^. Previous studies also demonstrated that exercise could promote calcium absorption, utilization and deposition and significantly increase estradiol and testosterone levels *in vivo*^40^. Thus, the combined therapy of NG + EX seemed to promote equilibrium between resorption and formation via the estrogen-like effect of naringin plus mechanical stimulation of exercise which seems to have an additive or synergistic effect on osteoblasts and osteoclasts.

Previous studies have reported doses of naringin (100, 40 and 20 mg/kg/day) treatment on the retinoic acid-induced osteoporosis rats for 14 days. The results showed that the naringin-treated rats were superior to the untreated rats in the bone weight index, the length and the diameter of the femur bone, the bone ash content, and the levels of calcium and phosphorus. The dose of 100 mg/kg of naringin demonstrated greater effects on BMD of the femur^9^. Pang *et al.* demonstrated that the dose rate at 400 mg/kg of naringin by gavage daily for 2 months increased BMD of trabecular-rich bone in OVX mice. The dose rate at 200 mg/kg/day and 400 mg/kg/day of naringin showed similar increased stress-strain indices at the distal femur and lumbar spine and increased biomechanical strength at tibia diaphysis in OVX mice^6^. Li *et al.* reported that different concentrations of naringin (60, 300, and 1500 mg/kg) by gavage daily for 2 months treatment^32^. The dose rate at 300 mg/kg of naringin was chosen based on a previously reported rat study because this medium dose (300 mg/kg) appeared to be the optimal dosage to reverse OVX-induced bone loss via increasing BMD, bone volume, and trabecular thickness^32^. Treatments were given orally for 60 days. Iwamoto *et al.* tested different intensities on the treadmill with ovariectomized adult rats. Moderate exercise was more efficient to increase BMD and bone strength of the tibia of the animals, while high-intensity exercise accelerated bone loss. Our training protocol was based on previous reports demonstrating that treadmill running is an osteogenic stimulus in rats^17^ and is considered moderate exercise. The current investigation served to evaluate whether the dose rate at 300 mg/kg of naringin plus moderate exercise may produced positive therapeutic effects on bone tissue.

The mechanism of postmenopausal osteoporosis affecting bone impairment has not been completely identified. Although NG + EX in this study produced stronger effects than either monotherapy on osteoporosis in OVX rats within 60 days, longer observation time is needed for evaluation of the long-term efficiency of combined treatment. Furthermore, the physiological condition is different between OVX rats and postmenopausal women, and the treatment effects of naringin and exercise in this study is limited by the use of the animal model. The safety and efficacy of a combined intervention of naringin and exercise therapy should be further addressed in human models to determine the efficacy of this comprehensive treatment for post-menopausal osteoporosis.

In conclusion, this study suggests that combined NG + EX held demonstrated greater effects on osteoporosis than either monotherapy in OVX rats. NG + EX produced the strongest effects on BMD, BV/TV, Tb.Th, MAR, and bone strength. NG + EX exerted locally increased OCN and decreased OCN levels in bone metabolism by serum tests. These results indicate that NG plus EX has an additive effect on osteoporosis associated with estrogen deficiency, and could potentially reduce the risk of fracture.

## Additional Information

**How to cite this article**: SUN, X. *et al.* The Effects of Combined Treatment with Naringin and Treadmill Exercise on Osteoporosis in Ovariectomized Rats. *Sci. Rep.* **5**, 13009; doi: 10.1038/srep13009 (2015).
